# Specificity and Strain-Typing Capabilities of Nanorod Array-Surface Enhanced Raman Spectroscopy for *Mycoplasma pneumoniae* Detection

**DOI:** 10.1371/journal.pone.0131831

**Published:** 2015-06-29

**Authors:** Kelley C. Henderson, Alvaro J. Benitez, Amy E. Ratliff, Donna M. Crabb, Edward S. Sheppard, Jonas M. Winchell, Richard A. Dluhy, Ken B. Waites, T. Prescott Atkinson, Duncan C. Krause

**Affiliations:** 1 Department of Microbiology, University of Georgia, Athens, GA, United States of America; 2 Pneumonia Response and Surveillance Laboratory, Centers for Disease Control and Prevention, Atlanta, GA, United States of America; 3 Department of Pathology, University of Alabama at Birmingham, Birmingham, AL, United States of America; 4 Department of Pediatrics, University of Alabama at Birmingham, Birmingham, AL, United States of America; 5 Department of Chemistry, University of Georgia, Athens, GA, United States of America; National Jewish Health, UNITED STATES

## Abstract

*Mycoplasma pneumoniae* is a cell wall-less bacterial pathogen of the human respiratory tract that accounts for > 20% of all community-acquired pneumonia (CAP). At present the most effective means for detection and strain-typing is quantitative polymerase chain reaction (qPCR), which can exhibit excellent sensitivity and specificity but requires separate tests for detection and genotyping, lacks standardization between available tests and between labs, and has limited practicality for widespread, point-of-care use. We have developed and previously described a silver nanorod array-surface enhanced Raman Spectroscopy (NA-SERS) biosensing platform capable of detecting *M*. *pneumoniae* with statistically significant specificity and sensitivity in simulated and true clinical throat swab samples, and the ability to distinguish between reference strains of the two main genotypes of *M*. *pneumoniae*. Furthermore, we have established a qualitative lower endpoint of detection for NA-SERS of < 1 genome equivalent (cell/μl) and a quantitative multivariate detection limit of 5.3 ± 1 cells/μl. Here we demonstrate using partial least squares- discriminatory analysis (PLS-DA) of sample spectra that NA-SERS correctly identified *M*. *pneumoniae* clinical isolates from globally diverse origins and distinguished these from a panel of 12 other human commensal and pathogenic mycoplasma species with 100% cross-validated statistical accuracy. Furthermore, PLS-DA correctly classified by strain type all 30 clinical isolates with 96% cross-validated accuracy for type 1 strains, 98% cross-validated accuracy for type 2 strains, and 90% cross-validated accuracy for type 2V strains.

## Introduction

The cell wall-less prokaryote *Mycoplasma pneumoniae* is a major cause of respiratory disease in humans, accounting for 20% to 40% of all community acquired pneumonia (CAP). *M*. *pneumoniae* is the leading cause of CAP in older children and young adults, while the incidence of infection in the very young and the elderly is on the rise [1–5]. For adults alone the annual economic burden of CAP is > $17 billion [6]. Macrolide resistance is a growing concern, particularly in children [5], and extra-pulmonary sequelae occur in up to 25% of infections. Finally, evidence continues to indicate a contributing role for *M*. *pneumoniae* infection in the onset, exacerbation, and recurrence of asthma [5].

An area of growing interest is the role of *M*. *pneumoniae* strain type in pathogenesis and disease epidemiology. Genetic diversity is relatively limited among *M*. *pneumoniae* strains, which can be categorized into two major groups (type 1 or type 2) based on variation within sequence of the *P1* (MPN141) gene, although variant strains of the two are increasingly more common [7]. The P1 protein is an important virulence factor and immunogen in *M*. *pneumoniae* infection [8–10]. P1 must complex with several other proteins in order to localize to the tip of the terminal organelle, where it mediates receptor binding for attachment to the respiratory epithelium, an essential step in successful colonization of the airways [9, 11]. Variation in the *P1* gene sequence is used to distinguish between type 1 and type 2 strains of *M*. *pneumoniae*, but little is known about phenotypic differences arising from this genetic variation. Perhaps notable in this regard is the periodicity of type-switching that occurs between the two major genotypes in regular patterns of four to seven years [12].

*M*. *pneumoniae* infection is transmitted through aerosolized respiratory secretions and spreads slowly but efficiently through close living quarters, with incubation periods up to three weeks [13, 14]. Symptoms tend to be non-descript, often with complex and variable clinical presentations, which makes definitive diagnosis challenging [2, 6, 15]. As a result, diagnosis is often presumptive and relies heavily on the combination of physical findings and the elimination of other possible causes [4, 5, 14]. The success rate for laboratory culture is poor, even for experienced labs, while serologic testing, historically considered the foundation for diagnosis of *M*. *pneumoniae* infection, has limited sensitivity and specificity, a high tendency for false-negatives, requires paired sera resulting in retrospective diagnosis, and must often be paired with another diagnostic method [2, 4, 5, 10, 14]. Of the currently existing alternatives, the most efficient means for detection is quantitative polymerase chain reaction (qPCR). At present, the only FDA-approved tests for the clinical detection of *M*. *pneumoniae* are the Illumigene automated detection system (Meridian Bioscience, Inc., Cincinatti, Ohio) and the FilmArray Respiratory Panel (BioFire Diagnostics Inc., Salt Lake City, Utah). The Illumigene platform uses loop-mediated isothermal amplification and is capable of detecting *M*. *pneumoniae* in both throat and nasopharyngeal swab specimens with a high degree of sensitivity and specificity. The FilmArray Respiratory Panel employs nested, multiplex qPCR with endpoint melt curve analysis on nasopharyngeal swabs to test for 21 different viral and bacterial respiratory pathogens, and is capable of detecting *M*. *pneumoniae* as low as 30 colony-forming units (CFU)/ml [16]. The current standard for *M*. *pneumoniae* genotyping is PCR-restriction fragment length polymorphism, but can also be done by nested PCR and sequencing, multilocus variable-number tandem-repeat analysis, or by qPCR and high resolution melt curve analysis [15, 17–19]. These methods for detection and genotyping exhibit high sensitivity and specificity for all known strain variants, can allow for detection in the early stages of infection, and can be performed in hospitals and reference laboratories [2, 4, 5]. However, the requirement for separate tests for detection and genotyping, as well as the cost, complexity, and expertise required, limits the practicality for widespread, point-of-care use [2, 4–6, 14]. These limitations create a critical barrier to the accurate and timely diagnosis of *M*. *pneumoniae* infection and epidemiological tracking, and a rapid, simple, diagnostic platform capable of simultaneous detection and genotyping would greatly improve the control of *M*. *pneumoniae* disease.

Vibrational spectroscopy has an inherent biochemical specificity that led to its consideration as a next-generation platform for the rapid detection, characterization, and identification of infectious agents [20–23]. Raman spectroscopy in particular has several advantages for application to biological samples, including narrow bandwidths, good spatial resolution, and the ability to analyze aqueous samples due to the absence of interference by water molecules [20, 21, 24]. Furthermore, Raman spectra provide detailed structural information on the chemical composition of a sample and can serve as a characteristic molecular fingerprint for pathogen identification [23, 24]. Despite these advantages, standard Raman spectra are inherently limited by weak signals for detection. As a result, the application of traditional Raman spectroscopy for biosensing applications was impractical and inefficient [13, 21, 24] until the discovery that sample adsorption onto nanoscopically roughened metallic surfaces results in significant enhancements in Raman signal and spectral intensity [23–25]. This enhancement, by factors up to 10^14^-fold, is attributed to the increased electromagnetic field for molecules in close proximity to the metallic surface [20, 21]. Surface-enhanced Raman spectroscopy (SERS) retains the advantages of standard Raman spectroscopy, in addition to markedly improved sensitivity, allowing for considerable success at whole organism molecular fingerprinting [20, 24, 26, 27].

Inconsistency and lack of reproducibility in the preparation of SERS-active substrates has hindered its widespread use for biosensing applications [20, 21, 24]. However, highly ordered silver nanorod array (NA) substrates fabricated using oblique angle deposition (OAD) yield consistent SERS enhancement factors of around 10^8^, with less than 15% variation between substrate batches [21]. The reproducibility of NA-SERS substrates can be improved further when patterned into a multiwell format with polydimethylsiloxane (PDMS) [20]. The highly reproducible detection capabilities of NA-SERS have been well demonstrated for multiple infectious agents, including RSV, rotavirus, influenza, HIV, adenovirus, SARS coronavirus, and *M*. *pneumoniae* [13, 22, 28–30].

Hennigan et al. described an NA-SERS-based platform capable of detecting *M*. *pneumoniae* with statistically significant sensitivity and specificity in both simulated and true clinical throat swabs, with the potential to detect and type *M*. *pneumoniae* within a single test [13]. We recently determined the sensitivity of NA-SERS for *M*. *pneumoniae* detection to be < 1 genome equivalent (cell/μl) qualitatively, and to have a quantitative multivariate detection limit of 5.3 ± 1 cells/μl [31]. Initial evaluation of this biosensing platform’s capabilities indicates the potential for application as a next-generation diagnostic tool for the clinical detection of *M*. *pneumoniae*, but a more comprehensive analysis is needed prior to proceeding with clinical validation. In the present study we further explored the specificity of NA-SERS for *M*. *pneumoniae* detection with a panel of 30 *M*. *pneumoniae* isolates collected from representative global outbreaks and spanning clinically relevant genotypes. Furthermore, since NA-SERS has inherent biochemical specificity, we analyzed a panel of 12 other human commensal and pathogenic mycoplasmas to demonstrate that this biosensing platform could distinguish *M*. *pneumoniae* from its clinically relevant closest phylogenetic relatives. Finally, we evaluated the ability of the NA-SERS platform to correctly type the 30 *M*. *pneumoniae* clinical isolates relative to known reference strains of *M*. *pneumoniae*.

## Methods

### Preparation of *M*. *pneumoniae* controls and clinical isolates for SERS analysis

Wild type *M*. *pneumoniae* reference strains M129 (type 1) and FH (type 2) were grown, harvested, and prepared at the University of Georgia (UGA) for this study. A panel of 30 additional clinical isolates consisting of 13 type 1 strains, 11 type 2 strains, and six type 2 variant strains were grown, harvested, and prepared for SERS and quality control analysis at the Pneumonia Response and Surveillance Laboratory at the Centers for Disease Control and Prevention (CDC) in Atlanta, Georgia. *P1* genotype groups were determined by the Pneumonia Response and Surveillance Laboratory at the CDC in Atlanta, GA using DNA sequence analysis, qPCR in combination with high resolution melt curve analysis, and RFLP sequencing analysis. All mycoplasma isolates and controls were cultured in SP4 medium [2, 30] in tissue culture flasks with a 1μl/ml inoculation and incubated at 37°C. Strains grown at UGA were harvested at log phase when the phenol red indicator turned an orange color upon reaching a pH of ~6.5. Strains grown at the CDC were harvested 14 days from the date of inoculation to ensure adequate growth for all isolates. At time of harvest, the spent growth medium was decanted for each flask and 0.1× volume of sterile PBS (pH 7.2) was added to wash the adherent mycoplasmas. The PBS wash was then decanted and the PBS wash repeated 3× before the cells were scraped into 1 ml sterile PBS. Cells were then syringe-passaged 10× with a 25 gauge needle and aliquots made for determination of protein content, plating on PPLO agar [32] for CFU determination (for select isolates and controls), DNA extraction for genome equivalent determination, and SERS analysis.

*M*. *pneumoniae* samples for SERS analysis were syringe-passaged 10× with a 25-gauge needle to disperse clumps, fixed with the addition of one volume of 8% formaldehyde in sterile PBS (pH 7.0), and stored at 4°C. Growth medium negative control samples were prepared in parallel under the same conditions as the *M*. *pneumoniae* reference strains as described previously [31]. At the time of SERS analysis, mycoplasma and medium-only negative control samples were diluted in sterile DI water to a concentration of 10^5^ cells/μl, which falls within the SERS detectable range for *M*. *pneumoniae* and was found to be dilute enough to ensure that the spectra adequately represent SERS Raman spectra arising from the Ag nanorod substrate [31]. Samples were loaded onto the NA-SERS substrate immediately following this dilution.

### Preparation of non-*M*. *pneumoniae* human commensal and pathogenic species for NA-SERS analysis

Twelve human commensal and pathogenic *Mollicutes* species closely related [33] to *M*. *pneumoniae* were grown and harvested at the University of Alabama at Birmingham (UAB). These included: *Acholeplasma laidlawii* (ATCC 23206), *Mycoplasma amphoriforme* (A39 M6123; provided by M. Balish, Miami University of Ohio), *Mycoplasma fermentans* (ATCC 19989), *Mycoplasma genitalium* (ATCC 49897), *Mycoplasma hominis* (ATCC Mh132), *Mycoplasma orale* (ATCC 23714), *Mycoplasma penetrans* (UAB reference strain collection, year 1995), *Mycoplasma pirum* (ATCC 25960), *Mycoplasma salivarium* (ATCC 23064), *Mycoplasma spermatophilum* (ATCC 49695), *Ureaplasma parvum* (Up1; ATCC 27813), and *Ureaplasma urealyticum* (Uu11; ATCC 33695). *Mycoplasma buccale*, *Mycoplasma lipophilum*, and *Mycoplasma faucium* were originally intended to be included in the panel, but attempts at culturing these organisms were unsuccessful. For each culture, 500 μl to 1 ml of stock culture was inoculated into approximately 30 ml of SP4, Hayflick’s, or 10B depending on the individual species’ growth requirements, and incubated until the pH indicator changed color, indicative of microbial growth and utilization of the metabolic substrate in the media, i.e., glucose, arginine, or urea. At the time of harvest the cells and spent media were poured into 50 ml polycarbonate tubes and centrifuged at 8,000 RPM for 15 min, except for *Ureaplasma* species, which were centrifuged for 1 hr. The supernatants were decanted and the pellets suspended in 30 ml sterile PBS. The cells were washed by centrifugation at 8,000 RPM for 15 min as above, or 10,000 RPM for 1 hr for *Ureaplasma* species. The supernatants were then decanted and the pellets suspended in 1 ml sterile PBS, transferred to a 1.5 ml vial, and centrifuged at 14,000 RPM for 20 min. The supernatants were again decanted and the pellets suspended in 1 ml sterile PBS and syringe-passaged using a 26-gauge needle to disperse clumps. Aliquots were made for spotting onto a blood agar plate to test for contamination, and plating for CFU and color-changing unit (CCU) determination. Two 400 μl aliquots for each were centrifuged at 14,000 RPM for 20 min, the supernatant was removed, and the pellets were frozen for shipment to UGA, where they were stored at -80°C.

For SERS and quality control analysis, cell pellets were suspended in 1 ml sterile PBS (pH 7.2) and syringe-passaged 10× with a 25 gauge needle to disperse clumps. Aliquots were then made for DNA extraction and genome equivalent determination, protein assay, and NA-SERS analysis. SERS samples were prepared by fixing 500 μl of suspended cells with 500 μl of 8% formaldehyde in sterile PBS (pH 7.0), and stored at 4°C until time of SERS analysis. At that time the samples were diluted in sterile DI water to a concentration of 10^3^ to 10^4^ cells/μl and then immediately loaded onto the NA-SERS substrate. A growth medium only negative control and *M*. *pneumoniae* strain M129 samples were prepared as described above for comparison.

### Preparation of samples for determination of protein content and genome equivalents

All samples were analyzed for protein content via the Bicinchoninic acid assay [34]. DNA was extracted by the QIAamp DNA Blood Minikit (Qiagen, Valencia, CA) using the blood and body fluids protocol, including RNase A treatment. 200 μl of sample were used for DNA extraction, with a final elution volume of 200 μl for use to quantify DNA content and genome equivalents. Genomic DNA concentration and absorbance measurements for the Bicinchoninic acid assay were performed on a NanoDrop instrument (Model ND-1000, Thermo Scientific, Wilmington, DE) using software V3.5.2. Genome equivalents of *M*. *pneumoniae* samples were calculated from the DNA concentration obtained from this analysis and using the previously determined mass of the *M*. *pneumoniae* genome, 5.3x10^7^ Daltons [35]. Genome equivalents for all non-*M*. *pneumoniae* samples were determined from DNA concentrations obtained from this analysis and a genome mass calculated for this study based on published genome lengths and known G+C contents from the GenBank database.

### NA-SERS and chemometric analysis

NA-SERS substrates were prepared by OAD as described [21, 29, 36, 37]. Prior to their use, substrates were cleaned for 5 min in an Ar+ plasma using a plasma cleaner (Model PDC-32G, Harrick Plasma, Ithaca, NY) to remove any surface contamination [38] and then patterned into 40 3mm diameter PDMS-formed wells. 1,2-bis(4-pyridyl)ethylene (BPE; 10^-4^ Molar in methanol) was used as an external control to ensure consistency between substrates. Raman spectra were acquired using a Renishaw inVia Reflex multi-wavelength confocal imaging microscope (Hoffman Estates, IL). A Leicha apochromatic 5× objective (NA 0.12) illuminated a 1265 μm^2^ area on the substrate, which allows spatial averaging and minimizes the effect of potential random hot spots. A 785-nm near-infrared diode laser (Renishaw) operating at 10% power capacity (28 mW) provided the incoming radiation, and spectra were collected in 3 10-sec acquisitions. An internal silicon standard measurement was obtained at the beginning of each SERS analysis as an internal control for instrument performance.

All samples were applied in duplicate to the NA substrates at the concentrations specified, in a volume of 1 μl per well, and allowed to dry overnight. Spectra were collected from five random locations within each sample spot for analysis, for a total of 10 spectra per sample, and *M*. *pneumoniae* reference strain and growth medium controls were independently prepared and analyzed for each substrate. Two wells per substrate were intentionally left blank to obtain a background SERS reading on the naked nanorod substrate only. A total of three separate NA substrates were used for these experiments: two for analysis of *M*. *pneumoniae* isolates with n = 390 spectra, and one for analysis of other human and commensal *Mollicutes* species with n = 150 spectra, resulting in a total of n = 540 spectra. Spectra were deliberately collected from multiple locations within a single substrate, as well as from multiple substrates, to ensure any inherent variance present in the substrates did not impact the results. Raman spectra between 400–1800 cm^-1^ were acquired using Renishaw’s WiRE 3.4 software. Instrument settings were optimized to maximize signal and minimize saturation or sample degradation arising from laser stimulation [13, 20].

Raman spectra were first averaged using GRAMS32/A1 spectral software package (Galactic Industries, Nashua, NH) in order to assess signal-to-noise quality, and baseline-corrected using a concave rubberband algorithm which performed 10 iterations on 64 points to aid in preliminary evaluation of the spectra and peak assignment (OPUS, Bruker Optics, Inc., Billerica, MA). Chemometric analysis was carried out with MATLAB version 7.10.0 (The Mathworks, Inc., Natick, MA) using PLS-Toolbox version 7.5.1 (Eigenvector Research Inc., Wenatchee, WA). Raw spectra were pre-processed using the 1^st^ derivative of each spectrum and a 15-point, 2^nd^ order polynomial Savitsky-Golay algorithm. Each dataset was then vector-normalized and mean-centered. Due to the inherently complex nature of spectral data, multivariate statistical analysis of the datasets was performed using principal component analysis (PCA) and partial least squares-discriminatory analysis (PLS-DA), using the PLS Toolbox software. Unless otherwise specified, all PLS-DA models were cross-validated using a Venetian blinds algorithm with 10 data splits. All PLS-DA models in this study, excluding those for individual sample analysis, were generated using between 110–495 total spectra per model.

## Results and Discussion

### Detection of *M*. *pneumoniae* clinical isolates

**Fig 1:**
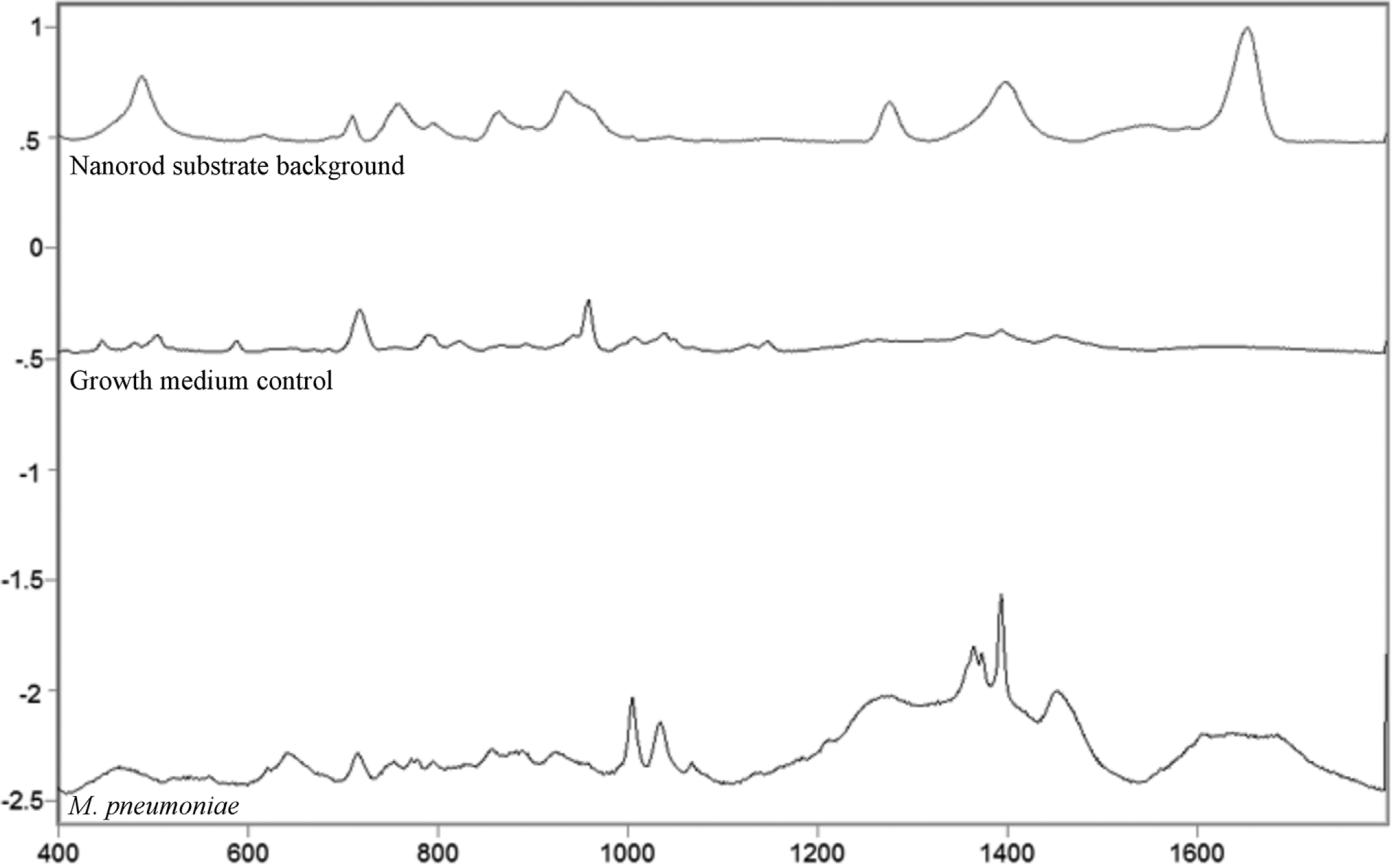
Averaged, baseline-corrected, and normalized SERS spectra for the nanorod substrate, growth medium control, and *M*. *pneumoniae* reference strain controls and clinical isolates, as indicated. Raw spectra of the three sample classes were averaged, baseline-corrected, and normalized using GRAMS32/A1 spectral software package (Galactic Industries, Nashua, NH). For the nanorod substrate background class, n = 20; for the growth medium control class, n = 20; and for the *M*. *pneumoniae* class, n = 350.

We analyzed 32 clinical isolates, including reference strains M129 (type 1) and FH (type 2), alongside a growth medium control prepared in parallel with the *M*. *pneumoniae* samples. Full details regarding isolate origin and year, P1 genotype, macrolide susceptibility, protein and DNA content, and genome equivalents for *M*. *pneumoniae* strains are given in Table 1. CFU values were determined for both reference strains and six randomly chosen additional isolates to assess cell viability at time of fixation and ranged from 1x10^5^ to 1x10^7^ CFU/ml. Due to the propensity for mycoplasma cells to clump, a confounding factor in using CFU values as a metric for sample content is the potential discrepancy between CFU value and actual cell number, which can differ by as much as 10^3^-fold [39]. Therefore, protein content and genome equivalents were determined in order to better define the content of the samples at the concentration analyzed by SERS. These values fell within comparable ranges and were consistent with published values for bacterial cells [40]. Protein concentration per cell was higher for *M*. *pneumoniae* isolates harvested during stationary phase relative to those harvested during log phase (growth phase based on the color of the pH indicator in the SP4 medium), but no notable differences in genome equivalents or SERS spectra were observed between *M*. *pneumoniae* samples relative to growth phase at time of harvest (data not shown). Average SERS spectra of the nanorod substrate background, growth medium control, and *M*. *pneumoniae* samples are shown in Fig 1, with each class exhibiting a distinct band pattern, as expected.

**Table 1:** *M*. *pneumoniae* strain details.

Isolate	Location	Year isolated	Genotype^a^	Macrolide phenotype^b^	Protein content (μg/μl)	DNA content (ng/μl)	Genomic equivalents (cells/μl)	fg of protein / cell
E1	Egypt	2009	2	S	0.149	2.0	4.49x10^6^	26
E16	Egypt	2010	1	S	0.057	1.7	4.34x10^6^	11
K3	Kenya	2010	2	S	0.065	2.3	6.5x10^6^	10
K20	Kenya	2010	1	S	0.044	1.2	3.39x10^6^	12.9
G6	Guatemala	2010	1	S	0.078	2.6	7.36x10^6^	10.6
NM2	NM	2010	1	R	0.074	1.1	3.11x10^6^	23.8
RI1	RI	2011	2	S	0.071	1.5	4.24x10^6^	16.7
OR1	OR	2011	1	R	0.076	1.3	3.68x10^6^	20.7
WV1	WV	2011	2	S	0.094	1.9	5.34x10^6^	17.6
WV9	WV	2012	1	R	0.071	1.3	3.68x10^6^	19.3
FL1	FL	2012	1	S	0.098	2.3	6.5x10^6^	15
WI11	WI	2014	2V	S	0.085	1.6	4.53x10^6^	18.8
WI17	WI	2014	2V	S	0.047	1.8	5.09x10^6^	9.2
CO12	CO	2014	2V	S	0.077	1.6	4.53x10^6^	16.9
CO44	CO	2014	2V	R	0.073	1.3	3.68x10^6^	19.8
SA18	S. Africa	2013	2V	S	0.35	1.7	4.81x10^6^	72.8
SA19	S. Africa	2013	2	S	0.47	1.1	3.11x10^6^	151.0
SA22	S. Africa	2013	1	S	0.44	1.1	3.11x10^6^	141.5
1005	NY	1999	2	S	0.28	1.5	4.24x10^6^	66.0
1134	IN	1999	2	S	0.30	2.2	6.22x10^6^	70.8
988	Canada	1992	1	S	0.12	2.0	5.65x10^6^	19.3
678	Denmark	1962	1	S	0.12	3.5	9.9x10^6^	12.1
682	Denmark	1988	2	S	0.16	2.1	5.94x10^6^	26.9
983	SC	1988	2	S	0.16	1.6	4.53x10^6^	35.3
386	TX	1994	2	S	0.10	2.9	8.2x10^6^	12.1
519	CA	1995	2	S	0.28	1.5	4.24x10^6^	63.6
GA1	GA	2012	1	S	0.22	3.5	9.9x10^6^	22.2
GA3	GA	2012	2V	S	0.46	3.3	9.33x10^6^	49.3
IL1	IL	2012	1	R	0.12	1.7	4.81x10^6^	24.9
IL2	IL	2012	1	R	0.10	1.8	5.09x10^6^	19.6
M129	N/A	N/A	1	S	0.169	4.1	1.13x10^7^	14
M129^c^	N/A	N/A	1	S	0.168	4.3	1.20x10^7^	14
FH	N/A	N/A	2	S	0.046	2.1	5.94x10^6^	7.7
FH^c^	N/A	N/A	2	S	0.046	2.2	6.30x10^6^	7.3

^a^ *M*. *pneumoniae P1* genotype groups based upon DNA sequence analysis performed by the Pneumonia Response and Surveillance Laboratory at the CDC in Atlanta, GA.

^b^ S, susceptible; R, resistant

^c^ Two independent preparations of M129 and FH were examined

N/A = information not available

**Fig 2:**
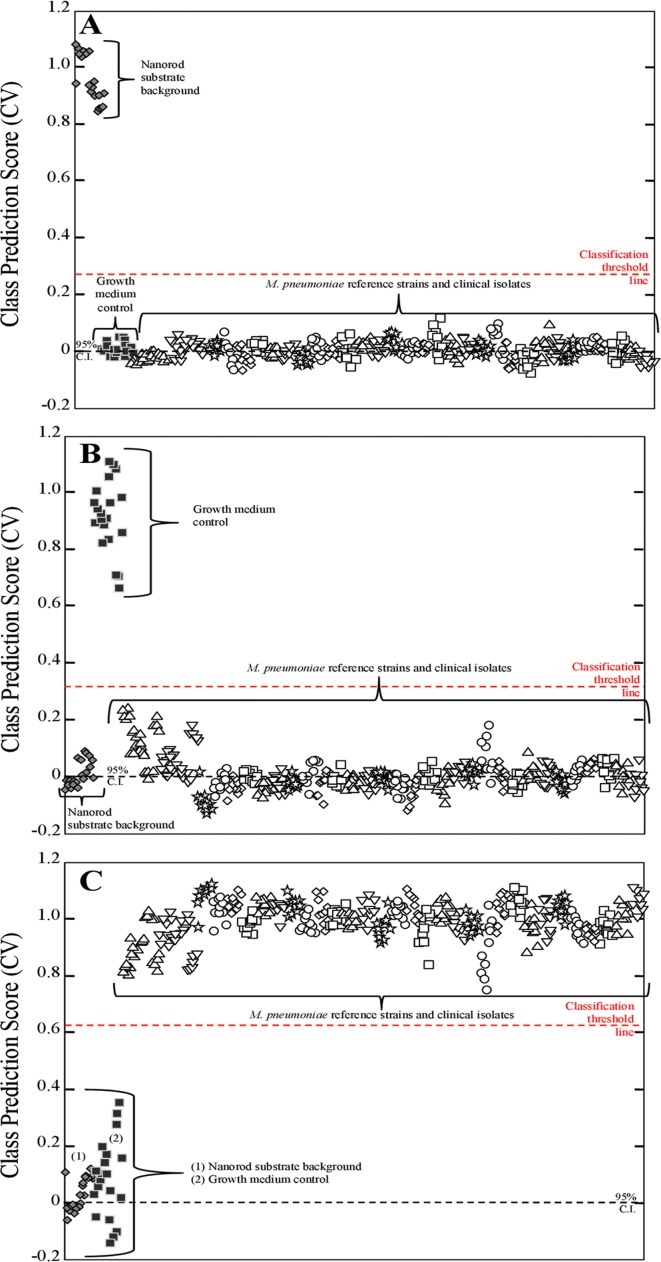
PLS-DA of 32 *M*. *pneumoniae* clinical isolates, including reference strains M129 and FH. Each panel represents a cross-validated class prediction score for **(A)** class 1, substrate background; **(B)** class 2, growth medium control; and **(C)** class 3, all *M*. *pneumoniae* strains. For panels A-C, each individual shape represents a single pre-processed NA-SERS spectrum. The substrate background spectra are represented by gray diamonds, the growth medium control spectra by solid black squares, and the *M*. *pneumoniae* spectra by open shapes that differ by cluster to indicate the different individual strains and isolates. The red-dotted line indicates the classification threshold line for positive class prediction, and the black-dotted line indicates the 95% confidence interval. Cross-validated sensitivity, specificity, and class error for the panels were as follows: **(A)** nanorod substrate background: 1.00, 1.00, and 0, respectively; for **(B)** growth medium control: 1.00, 1.00, and 0, respectively; and for **(C)** *M*. *pneumoniae*: 1.00, 1.00, and 0, respectively. Cross-validated statistics were obtained using Venetian blinds with 10 data splits to represent the prediction performance of the PLS-DA model for *M*. *pneumoniae* detection.

PLS-DA was applied here to determine statistically significant detection of *M*. *pneumoniae* by NA-SERS. PLS-DA is a full-spectrum, multivariate, supervised statistical method whereby prior knowledge of classes is used to yield more robust discrimination by minimizing variation within classes while emphasizing latent variables arising from spectral differences between classes [41, 42]. A PLS-DA model was generated to discriminate between three classes: the nanorod substrate background (Fig 2A); the growth medium control (Fig 2B); and all *M*. *pneumoniae* strains (Fig 2C). The inclusion of substrate background and growth medium controls allowed us to ensure that any differences in growth medium and nanorod background signal within the substrate did not affect the ability of the model to discriminate between the presence or absence of *M*. *pneumoniae*. Two nanorod substrates were used for these experiments, with each containing duplicate wells of the bare nanorod substrate, independently prepared M129, FH, and growth medium controls, and 15 additional clinical isolates of *M*. *pneumoniae*. A total of n = 390 pre-processed NA-SERS spectra collected from both substrates were included in the model, consisting of 20 nanorod substrate background spectra, 20 growth medium control spectra, 25 M129 spectra, 25 FH spectra, and 10 spectra per additional clinical isolate. The cross-validated statistics for the model show that NA-SERS correctly classified all 32 clinical isolates as *M*. *pneumoniae* regardless of global origin, year isolated, genotype, or macrolide susceptibility phenotype, and distinguished them from the substrate background and the growth medium control with 100% cross-validated sensitivity and specificity.

### Differentiation of *M*. *pneumoniae* and 12 other human commensal and pathogenic *Mollicutes* species

A critical question for clinical detection platforms is specificity for the pathogen of interest, particularly in the context of other organisms potentially present in a clinical sample. SERS is a structure-based technique that generates a Raman fingerprint or barcode based on the unique molecular content of the sample, and as such, the most likely organisms to generate false positives would be those most closely resembling *M*. *pneumoniae* structurally. To evaluate the specificity of the NA-SERS biosensing platform, 12 human commensal and pathogenic *Mollicutes* species closely related to *M*. *pneumoniae* in *rpoB* β-subunit nucleotide and amino acid sequence phylogenies and 16S rDNA phylogeny were chosen for analysis alongside *M*. *pneumoniae* strain M129 and a growth medium control [33]. In order to best define the content of the sample at the concentration used for SERS, analyses were done to determine total protein and DNA content, the latter allowing calculation of genome equivalents based on known genome sizes and G+C content (Table 2). These fell within comparable ranges and were consistent with published values for bacterial cells [40].

**Table 2:** Quality control and sample information for *Mollicutes* species and *M*. *pneumoniae* control cultures.

Commensal organism	Genome Size (Bp)^a^	Protein content (μg/μl)	DNA content (ng/μl)	Genomic equivalents (cells/μl)	fg protein/cell	CFU/μl
*Acholeplasma laidlawii*	1,496,992	0.247	16.2	1.9x10^6^	130	4.55x10^5^
*Mycoplasma amphoriforme*	1,029,022	0.072	2.4	4.3x10^5^	167	ND
*Mycoplasma fermentans*	1,118,751	0.374	23.8	3.9x10^6^	95.9	2.6x10^6^
*Mycoplasma genitalium*	580,073	0.099	2.6	8.2x10^5^	121	1.75x10^4^
*Mycoplasma hominis*	665,445	0.454	10.7	2.9x10^6^	157	2.2x10^5^
*Mycoplasma orale*	710,549	0.114	8.1	2.1x10^6^	54.3	9.9x10^4^
*Mycoplasma penetrans*	1,358,633	0.813	19	2.6x10^6^	313	1.7x10^6^
*Mycoplasma pirum*	510,593	0.384	20	7.2x10^6^	53.3	3.85x10^6^
*Mycoplasma salivarium*	710,000	0.558	32.1	8.32x10^6^	67.1	1x10^5^
*Mycoplasma spermatophilum*	846,000	0.068	1.2	2.6x10^5^	261	ND
*Ureaplasma parvum*	727,289	0.068	1.7	4.3x10^5^	158	4.5x10^4^
*Ureaplasma urealyticum*	874,478	0.067	2	4.2x10^5^	159	5.6x10^3^
*Mycoplasma pneumoniae*	816,394	0.061	1	2.24x10^6^	27.2	8.4x10^4^

^a^Genome sizes obtained from published sources [35] and known G+C contents from the GenBank database

**Fig 3:**
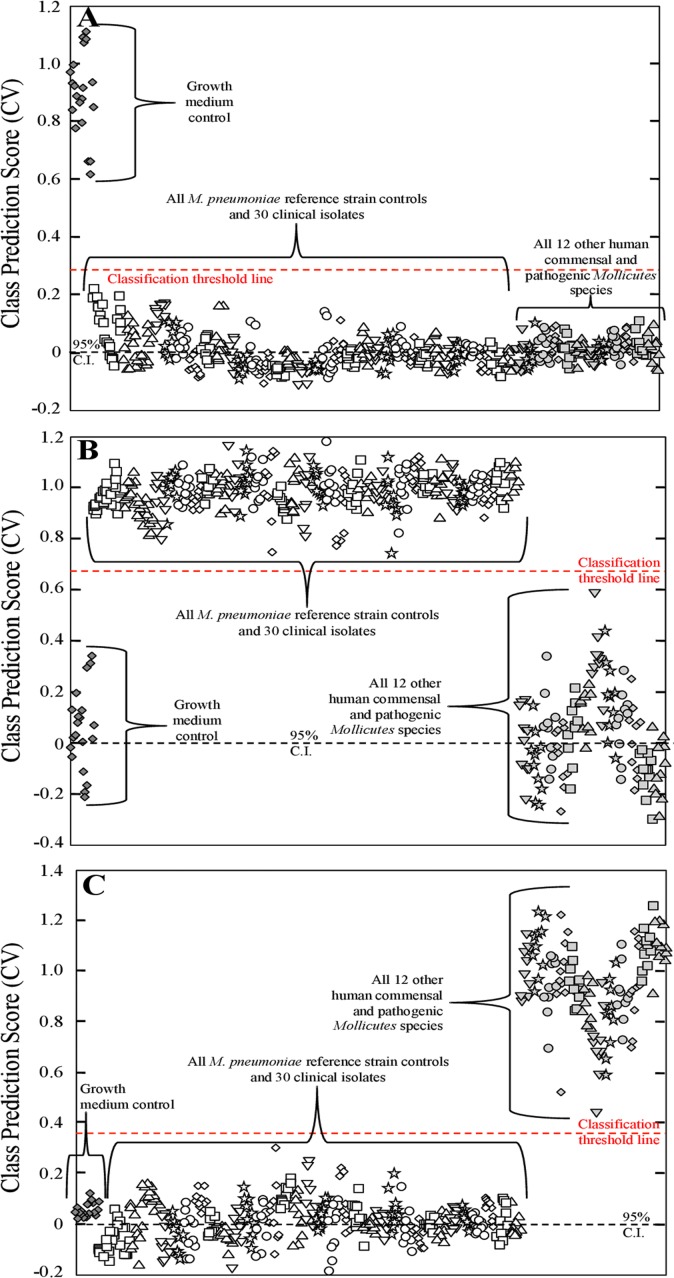
PLS-DA distinguishing *M*. *pneumoniae* strains from other human commensal and pathogenic *Mollicutes* species. Each panel represents a cross-validated class prediction score for **(A)** class 1, growth medium control; **(B)** class 2, all *M*. *pneumoniae* strains; and **(C)** class 3, all other human commensal and pathogenic *Mollicutes* samples. For panels A-C, each individual shape represents a single pre-processed NA-SERS spectrum. The growth medium control spectra are represented by gray diamonds, the *M*. *pneumoniae* spectra by open shapes that differ by cluster to indicate the different individual strains and isolates, and the human commensal and pathogenic *Mollicutes* species are represented by light gray shapes that differ by cluster to indicate the individual species. The red-dotted line indicates the classification threshold line for positive class prediction, and the black-dotted line indicates the 95% confidence interval. Cross-validated sensitivity, specificity, and class error for the panels were as follows: **(A)** growth medium control: 1.00, 1.00, and 0, respectively; for **(B)** All *M*. *pneumoniae* samples: 1.00, 1.00, and 0, respectively; and for **(C)** All 12 *Mollicutes* species: 1.00, 1.00, and 0, respectively. Cross-validated statistics were obtained using Venetian blinds with 10 data splits to represent the prediction performance of the PLS-DA model for *M*. *pneumoniae* detection.

Our goal here was to develop a PLS-DA model that distinguished *M*. *pneumoniae* from a panel of closely-related other *Mollicutes* species that might be found in humans. A total of n = 150 pre-processed NA-SERS spectra were collected on a single nanorod substrate consisting of n = 10 substrate background spectra, n = 10 growth medium control spectra, n = 10 *M*. *pneumoniae* spectra, and 10 spectra each per other *Mollicutes* species. An initial PLS-DA model was generated to discriminate between two classes, the nanorod substrate background and all other biological samples, which it did with 100% cross-validated sensitivity and specificity (data not shown). The purpose of this model was to ensure that the nanorod substrate background signal was significantly different than all other samples in order to exclude the background spectra from our future models. Once we determined that the nanorod substrate background class could be excluded, a second PLS-DA model was generated using the same spectra to distinguish among three classes; the growth medium control, *M*. *pneumoniae*, and the other *Mollicutes* species. This model had a total of n = 140 pre-processed NA-SERS spectra, consisting of n = 10 growth medium control spectra, n = 10 *M*. *pneumoniae* spectra, and 10 spectra each per other *Mollicutes* species. This model distinguished the three classes with 100% cross-validated sensitivity and specificity (data not shown). Upon the successful development of a PLS-DA model to distinguish between the growth medium control, *M*. *pneumoniae*, and all other *Mollicutes* species, a final PLS-DA model was generated using pre-processed NA-SERS spectra from all three nanorod substrates analyzed during these experiments. This model contained a total of n = 495 spectra, consisting of 25 growth medium control spectra, 25 M129 spectra, 25 FH spectra, 10 spectra each per other *M*. *pneumoniae* clinical isolates (30 isolates total), and 10 spectra each per other *Mollicutes* species (12 species total). This model was also categorized into three classes: the growth medium control (Fig 3A); all *M*. *pneumoniae* clinical isolates, including reference strains (Fig 3B); and all other *Mollicutes* species (Fig 3C). PLS-DA distinguished all *M*. *pneumoniae* strains from all 12 other human *Mollicutes* species and the growth medium control with 100% cross-validated sensitivity and specificity (Fig 3A–3C).

### *M*. *pneumoniae* typing capabilities of NA-SERS

A key advantage of NA-SERS for biosensing is the potential to detect and type an organism in a single test, especially of interest here since there is currently no existing platform capable of the simultaneous detection and typing of *M*. *pneumoniae*. To evaluate this capability we applied PLS-DA to the *M*. *pneumoniae* strain spectra above. Our panel of clinical isolates contained three distinct and clinically relevant genotypes of *M*. *pneumoniae*: 13 type 1 strains, 11 type 2 strains, and six type 2 variant (2V) strains. *M*. *pneumoniae* strains M129 (type 1) and FH (type 2) were used as reference strain controls, as they have been previously applied in this manner for evaluation of *M*. *pneumoniae* genotyping assays [19].

**Fig 4:**
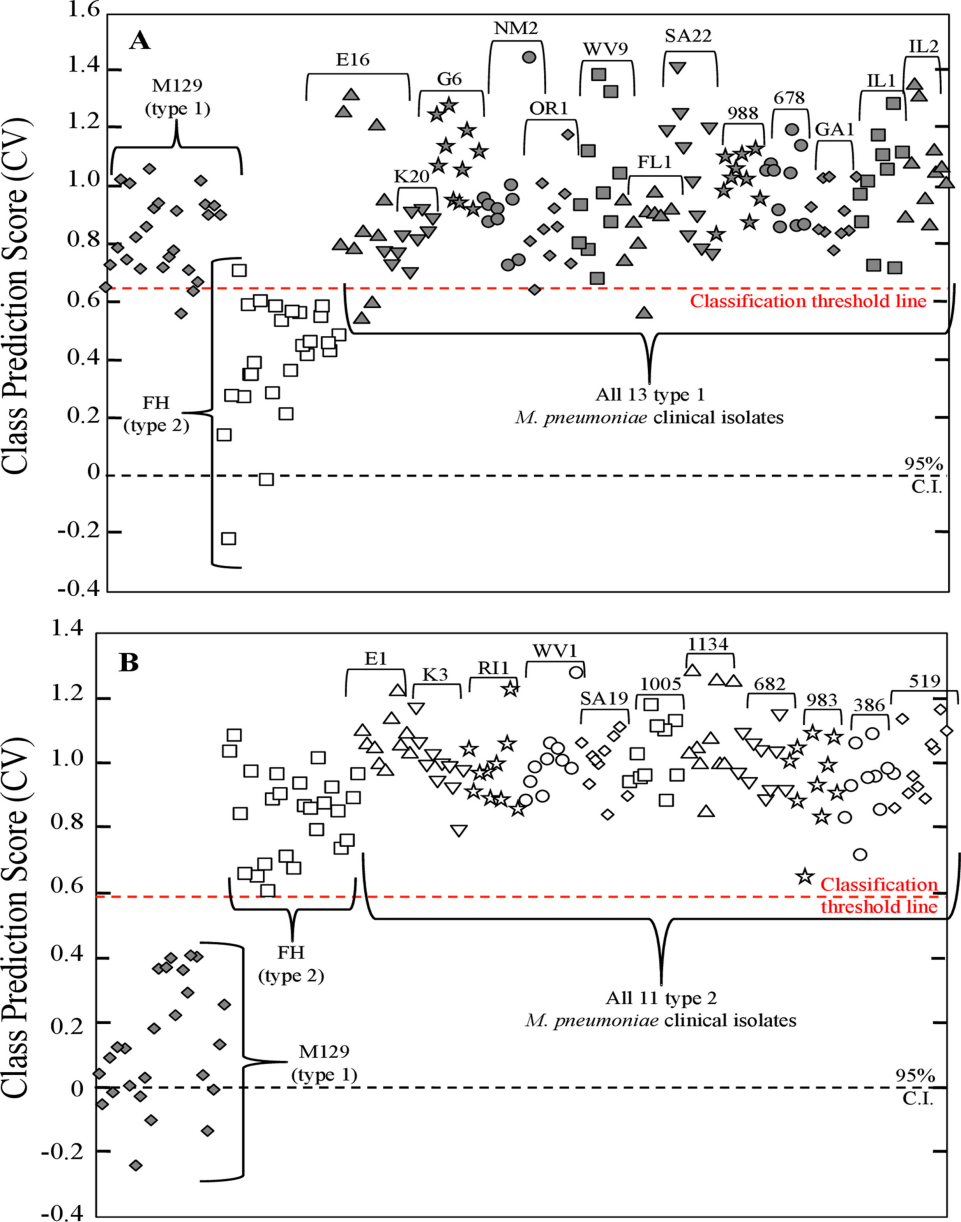
PLS-DA for NA-SERS typing of type 1 and 2 *M*. *pneumoniae* strains. Cross-validated class prediction scores for **(A)** all 13 type 1 clinical isolates, and **(B)** all 11 type 2 clinical isolates. For panels A and B, each individual shape represents a single pre-processed NA-SERS spectrum. *M*. *pneumoniae* type 1 reference strain control and other clinical isolates are represented by dark gray diamonds, while the type 2 reference strain control and other clinical isolates are represented by open shapes. Shapes differ by cluster to indicate the individual clinical isolates and samples, and the strain/isolate designation is indicated above the brackets for each cluster. The red-dotted line indicates the classification threshold line for positive class prediction, and the black-dotted line indicates the 95% confidence interval. The cross-validated sensitivity, specificity and class error were obtained using Venetian blinds with 10 data splits to represent the prediction performance of models for classification of **(A)** type 1 strains: 0.968, 0.96, and 0.04, respectively and for **(B)** type 2 strains: 1.00, 0.993, and 0.004, respectively.

For the type 1 strains a PLS-DA model was generated using 180 pre-processed NA-SERS spectra consisting of the 25 M129 spectra and the 25 FH spectra as controls, and all spectra from the 13 other type 1 clinical isolate samples (10 spectra per isolate, 130 total spectra). The model was built to discriminate between 2 classes, either type 1 or type 2. PLS-DA was able to correctly classify all other 13 type 1 strains with the type 1 reference strain with 96.8% sensitivity and 96% specificity (Fig 4A). For the type 2 strains a second PLS-DA model was generated using 160 pre-processed NA-SERS spectra consisting of the 50 type 1 and 2 reference strain control spectra and all spectra from the 11 other type 2 clinical isolate samples (10 spectra per isolate, 110 total spectra). This model was likewise built to discriminate between 2 classes, either type 1 or type 2. PLS-DA was able to correctly classify all 11 other type 2 isolates with the type 2 reference strain control with 99.3% sensitivity and 100% specificity (Fig 4B).

**Fig 5:**
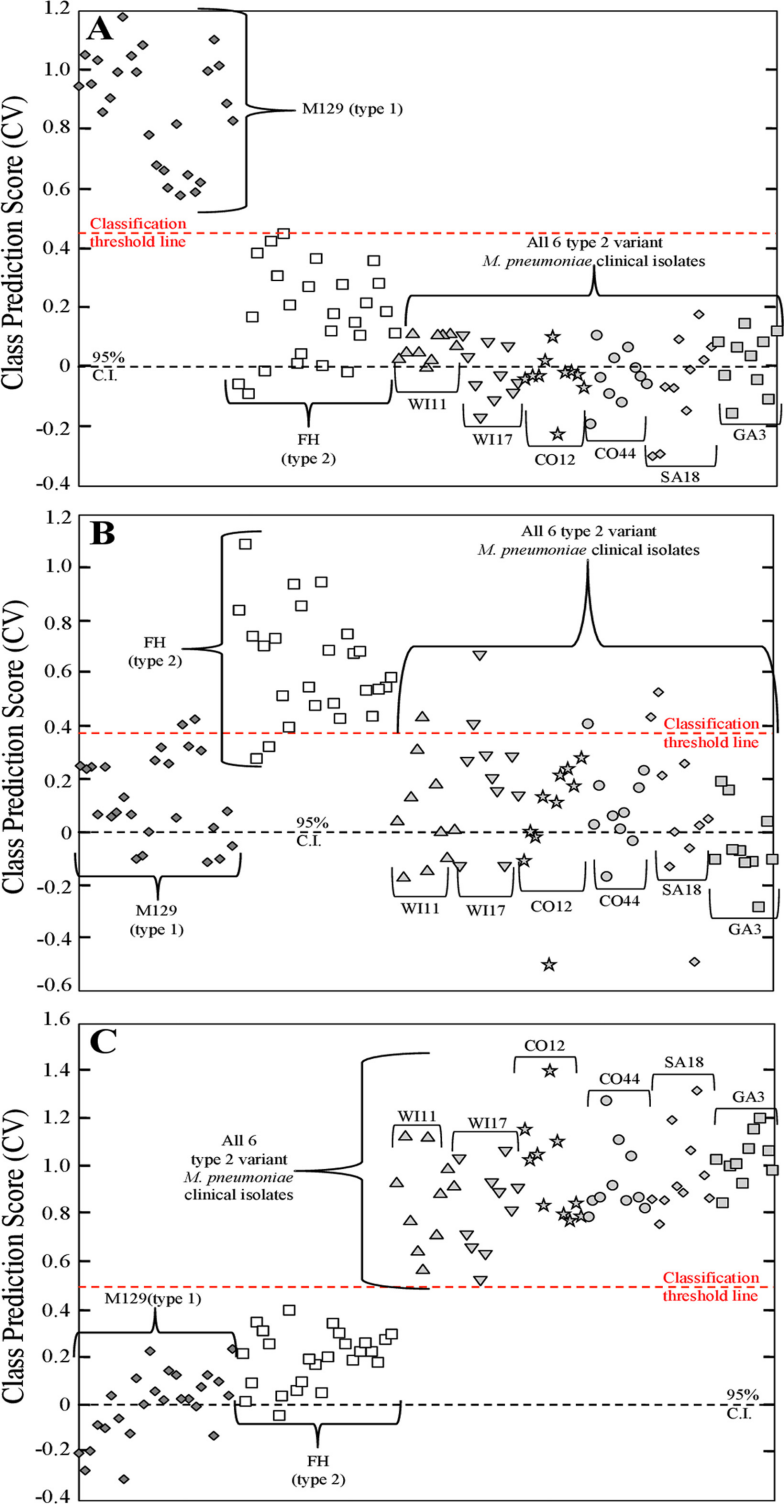
PLS-DA for NA-SERS typing of type 2V *M*. *pneumoniae* clinical isolates. Cross-validated class prediction scores for **(A)** class 1, the type 1 reference strain control; **(B)** class 2, the type 2 reference strain control; and **(C)** class 3, all six type 2V clinical isolates. For the panels A-C, each individual shape represents a single pre-processed NA-SERS spectrum. The *M*. *pneumoniae* type 1 reference strain control is represented by dark gray diamonds, the type 2 reference strain control is represented by open squares, and the type 2V clinical isolates are represented by light gray shapes. The light gray shapes differ by cluster to indicate the individual clinical isolates, and the strain/isolate designation is indicated above the brackets for each cluster. The red-dotted line indicates the classification threshold line for positive class prediction, and the black-dotted line indicates the 95% confidence interval. The cross-validated sensitivity, specificity and class error for panels A-C were obtained using Venetian blinds with 10 data splits to represent the prediction performance of **(A)** type 1 strains: 1.00, 0.988, and 0.006, respectively; for **(B)** type 2 strains: 0.92, 0.906, and 0.08, respectively; and for **(C)** type 2V strains: 1.00, 1.00, and 0, respectively.

For type 2V clinical isolates, a third PLS-DA model was generated using 110 pre-processed NA-SERS spectra consisting of the 50 type 1 and 2 reference strain control spectra and all spectra from the type 2V clinical isolate samples (10 spectra per isolate, 110 total spectra). However, this model was built to discriminate between 3 classes, type 1 reference strain control, type 2 reference strain control, or type 2V clinical isolate spectra. A third class was necessary for classification of this genotype, as existing methods are capable of identifying variant strains as unique from type 1 and 2 isolate strains [18], and as such for clinical purposes NA-SERS typing should be able to do the same. PLS-DA correctly classified the type 1 reference strain control as distinct from the type 2 control and the type 2V clinical isolates with 100% cross-validated sensitivity and 98.8% cross-validated specificity (Fig 5A). Furthermore, PLS-DA distinguished the type 2 reference strain control from the type 1 control and 2V clinical isolates with a cross-validated sensitivity and specificity of 92% and 90.6%, respectively (Fig 5B). Lastly, PLS-DA correctly classified all six type 2V strains as distinct from the type 1 and 2 reference strain controls with 100% cross-validated sensitivity and specificity (Fig 5C). The drop in sensitivity and specificity observed for the type 2 reference strain control is likely due to the fact that these are variant strains of the type 2 parent strain, and variant strains tend to be more similar to their respective parent strains genetically than either are to the opposite strain type [18, 37].

To further evaluate the strain typing capabilities of NA-SERS, PLS-DA models were generated using the M129 and FH reference strains alongside each clinical isolate individually. Thirty PLS-DA models were built using the 25 type 1 M129 spectra and 25 type 2 FH spectra as reference strain control classes, and 10 clinical isolate spectra treated as an unknown class. For type 1 and 2 clinical isolates, two categories were used for cross-validation of the model, while for type 2V isolate strains, three categories were incorporated to cross-validate the model, as described above. For all clinical isolate types the model was cross-validated by using a Venetian blinds algorithm with seven data splits. These PLS-DA models were incorporated to simulate a potential strategy for future application of NA-SERS for *M*. *pneumoniae* genotyping wherein known strain type controls are used to predict the genotype of an unknown clinical sample. Full cross-validated statistics for all 30 PLS-DA models are given in Table 3 for all type 1 and 2 clinical isolates, and Table 4 for all type 2V clinical isolates. Overall, PLS-DA performance was consistent with the models shown in Figs 4 and 5. The only notable difference in performance was a decrease in cross-validated specificity in the individual modeling for type 1 clinical isolates K20, NM2, and FL1, but this likely arises due to the decreased sample size (n = 60) used to build the individual PLS-DA models.

**Table 3:** Cross-validated PLS-DA modeling statistics for the prediction performance for NA-SERS typing of individual type 1 and 2 *M*. *pneumoniae* clinical isolates.

Isolate	P1 Type	CV Sensitivity	CV Specificity	CV Class Error
E16	1	0.943	0.92	0.06
K20	1	1	0.84	0.08
G6	1	1	0.92	0.04
NM2	1	1	0.88	0.06
OR1	1	0.914	0.92	0.08
WV9	1	0.914	0.96	0.06
FL1	1	0.971	0.84	0.09
SA22	1	0.971	1	0.01
988	1	0.971	1	0.01
678	1	1	1	0
GA1	1	1	1	0
IL1	1	1	1	0
IL2	1	0.971	1	0
E1	2	1	0.971	0.01
K3	2	1	1	0
RI1	2	1	1	0
WV1	2	1	1	0
SA19	2	1	1	0
1005	2	1	1	0
1134	2	1	0.971	0.01
682	2	1	1	0
983	2	1	1	0
386	2	1	1	0
519	2	1	1	0

Two categories were used for cross-validation of the model, either type 1 or type 2. Clinical isolates were treated as an unknown class and cross-validated sensitivity, specificity, and class error were based on their classification prediction score with their respective reference strain control class. CV, cross-validated.

**Table 4:** Cross-validated PLS-DA modeling statistics for the prediction performance for NA-SERS typing of individual type 2V *M*. *pneumoniae* clinical isolates.

Isolate	P1 Type	CV Sensitivity	CV Specificity	CV Class Error
1: M129	1	1	0.971	0.01
2: FH	2	0.8	0.943	0.13
3: WI11	2V	1	0.98	0.01
1: M129	1	1	1	0
2: FH	2	0.88	0.943	0.08
3: WI17	2V	1	0.98	0.01
1: M129	1	1	0.971	0.01
2: FH	2	0.84	1	0.08
3: CO12	2V	1	1	0
1: M129	1	1	1	0
2: FH	2	0.92	0.971	0.05
3: CO44	2V	1	0.98	0.01
1: M129	1	1	1	0
2: FH	2	1	0.943	0.03
3: SA18	2V	1	1	0
1: M129	1	1	1	0
2: FH	2	0.96	1	0.02
3: GA3	2V	1	1	0

Three categories were incorporated to cross-validate the model: either type 1; type 2; or neither. Clinical isolates were treated as an unknown class and cross-validated sensitivity, specificity, and class error were based on their classification prediction score as neither type 1 or type 2 reference control strains (i.e. category 3).

**Fig 6:**
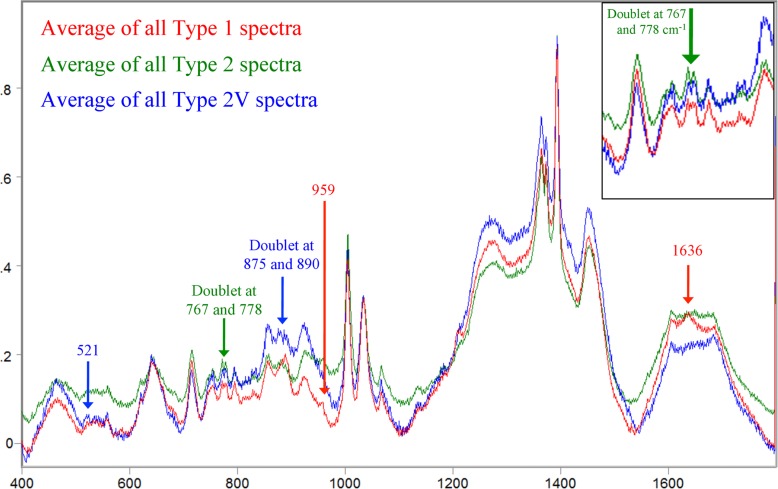
Comparison of averaged, baseline-corrected, and normalized SERS spectra for type 1, type 2, and type 2V genotypes. Raw spectra of all type 1 (n = 155), type 2 (n = 135), and type 2V (n = 60) clinical isolates and controls were averaged, baseline-corrected, and normalized using GRAMS32/A1 spectral software package (Galactic Industries, Nashua, NH). Red, average spectrum of all type 1 *M*. *pneumoniae* strains; green, average spectrum of all type 2 *M*. *pneumoniae* strains; blue, average spectrum of all type 2V *M*. *pneumoniae* strains. Peaks unique to a specific genotype of *M*. *pneumoniae* are indicated by arrows and identified above the spectral fingerprint. Type 1 peaks, red arrows; type 2 peaks, green arrows; and type 2V peaks, blue arrows. Inset at top right of image depicts zoomed-in view of the type 2 doublet at 767 and 778 cm^-1^.

Additionally, we compared averaged, baseline-corrected, and normalized spectra of all three genotypes to look for any differences in band pattern between the three genotypes that could be contributing to the classification capabilities demonstrated in the PLS-DA modeling (Fig 6). The majority of the spectral fingerprint was identical for all three strain types, which is to be expected since they are all the same species and classify as such in the PLS-DA models shown in Figs 2 and 3. However, several visible differences in band pattern were present in the spectra for each genotype of *M*. *pneumoniae*, which could account for the ability of NA-SERS to distinguish between the three genotypes with statistically significant sensitivity and specificity. The averaged type 1 spectrum had two unique peaks, one at 1636 cm^-1^ that does not appear in the averaged type 2 or 2V spectra, and one at 959 cm^-1^ which appeared as more distinct and shifted slightly right in the type 1 spectrum when compared to the type 2 spectrum, and did not appear in the type 2V spectrum. The averaged type 2 strain spectrum was very similar to the type 1 strain spectrum aside from the differences mentioned above and the presence of a doublet at 767 and 778 cm^-1^, which appeared as more distinct than that present in the type 2V spectrum and as a broad singlet in the type 1 spectrum. The averaged type 2V spectrum appeared to be the most distinct of the three, with a doublet at 875 and 890 cm^-1^ that appeared as a single peak at 890 in type 1 and 2 spectra, and a small peak at 521 that was also absent in type 1 and 2 spectra. While these spectral differences were extremely subtle, chemometric analysis is highly capable of discerning differences such as these with substantial discriminatory classification power [43].

Although little is known about the phenotypic effects of strain type beyond observable differences in biofilm formation [44], the genotypic differences between them are well characterized. Briefly, homologous recombination within the *p1* gene of repetitive element sequences located both in and outside the *p1* gene contributes to sequence variation between strain types [7]. Nucleotide and amino acid sequencing of 60 *M*. *pneumoniae* isolates indicates that trinucleotide short sequence repeats (SSR’s) coding for serine can be found in all strain types anywhere from 5–14 times, but appear to be most prevalent in type 1 strains [7]. Serine repeats may form a hinge structure and lead to downstream conformational differences in the P1 protein between the different strain types, which could potentially affect its interaction with the host as a surface antigen [45, 46]. In addition, 14 of the 60 isolates in the Zhao *et al*. study had point mutations in several variant strains corresponding to amino acid changes in P1 to glutamine, proline, asparagine, and isoleucine residues [7].

In our study, the peaks unique to the type 1 spectrum are commonly associated with vibrational mode bonds present in lysine (959 cm^-1^) and amide I or alpha helix (1636 cm^-1^) molecular structures [47–49]. The peaks unique to the type 2 spectral fingerprint located at 767 and 778 cm^-1^ are commonly associated with vibrational modes found in histidine, tryptophan, or carbohydrate bonds [48–50]. Finally, the peaks unique to the type 2V clinical isolates found at 521, 875 and 890 cm^-1^ are frequently associated with bonds present in histidine, tryptophan, ribose, indole, asparagine, methionine, glutamine, and S-S and C-C stretching vibrational modes [48–50]. Interestingly, the unique peaks present in the average spectra for the strain types analyzed in this study are predominately associated with protein backbone, amino acid residue, and DNA bond vibrations. Furthermore, spectral features in the averaged spectrum of the 2V variant strains are consistent with the point mutations identified in the Zhao *et al*. study [7], and our overall spectral interpretation of the averaged spectra for each strain type is consistent with what is known about the differences between strain types of *M*. *pneumoniae* infection.

### Unsupervised chemometric analysis of *M*. *pneumoniae* strain types and *Mollicutes* species

We applied PCA to supplement the PLS-DA modeling of sample spectra and evaluate the total variance present in our *M*. *pneumoniae* typing and other human commensal and pathogenic *Mollicutes* datasets. PCA is an unsupervised form of chemometric analysis which reduces the dimensionality of the dataset and facilitates establishing patterns and grouping of similar spectra without *a priori* knowledge of sample class [43]. PCA explains successively smaller proportions of the variance, with the first few principal components explaining the greatest percentage of total variance present in the dataset [51].

**Fig 7:**
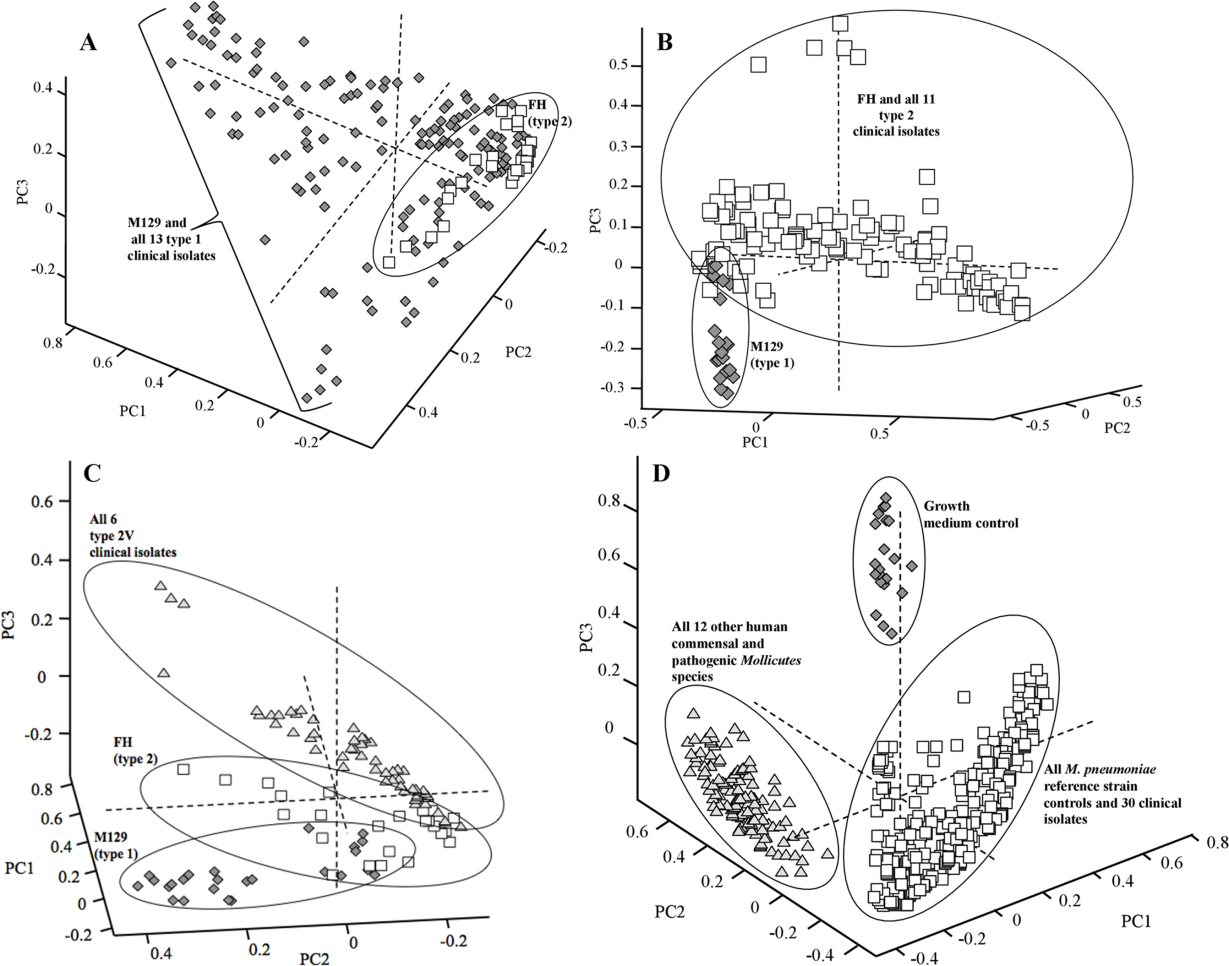
Principle component analysis of *M*. *pneumoniae* strain typing and other human commensal and pathogenic *Mollicutes* species. For all panels, each individual shape represents a single sample spectrum. PC scores plots of 1 vs. 2 vs. 3 of: **(A)** *M*. *pneumoniae* reference strains and all 13 other type 1 clinical isolates; **(B)** *M*. *pneumoniae* reference strains and all 11 other type 2 clinical isolates; **(C)** *M*. *pneumoniae* type 1 reference strain, type 2 reference strain, and all six type 2V clinical isolates; and **(D)** growth medium control, all *M*. *pneumoniae* strains, and all 12 other human commensal and pathogenic *Mollicutes* species. For panels A-C, dark gray diamonds represent type 1 sample spectra whereas open squares represent the type 2 sample spectra. In panel C, type 2V clinical isolate spectra are represented by light gray triangles. For panel D, growth medium control spectra are represented by dark gray diamonds, *M*. *pneumoniae* spectra by open squares, and all 12 other *Mollicutes* species by light gray triangles. For panels A-D, clustering of samples is indicated by black circles or brackets.

Pre-processed SERS spectra from *M*. *pneumoniae* reference strain type 1 and 2 controls and all other type 1 clinical isolates were used to generate a PCA plot comparing principle components 1, 2, and 3, which captured 54.3% of the total variance present in the 180 spectra used to build the model (Fig 7A). Type 2 control strain FH clustered in the bottom right corner, and the clustering pattern for all type 1 strains was predominately below and to the left, though some overlap between the two strain types was present. The PCA model of the type 1 clinical isolates supports the PLS-DA modeling of the spectra shown in Fig 4A.

A second PCA model was built using pre-processed SERS spectra (n = 160) consisting of type 1 and 2 reference strain controls and all other type 2 clinical isolates of *M*. *pneumoniae*. Principal components 1–3 captured 58.0% of the total variance and when plotted orthogonally showed a distinct separation between the type 1 reference strain control and all type 2 reference strain and other isolates, with very little overlap of clusters (Fig 7B). PCA modeling for the type 2 clinical isolate dataset was consistent with the PLS-DA modeling of the data shown in Fig 4B.

A third PCA model was built using the pre-processed SERS spectra from the type 2V clinical isolate dataset (n = 110). Principal components 1–3 captured 54.1% of the total variance and when plotted orthogonally showed distinctly separated clusters for the type 1 control, the type 2 control, and the type 2V clinical isolates, with some overlap present between the type 2 and type 2V clusters (Fig 7C). This clustering pattern further supports the PLS-DA classification performance shown in Fig 5.

Finally, a PCA model was built using the full *M*. *pneumoniae* and *Mollicutes* species dataset consisting of pre-processed spectra from all 3 nanorod array substrates (n = 495). Principal components 1–3 captured 50.1% of the total variance and when plotted orthogonally showed three distinctly separated clusters for growth medium control spectra, all *M*. *pneumoniae* spectra, and all other *Mollicutes* species spectra, with no overlap between clusters (Fig 7D). This supports the PLS-DA model of the data shown in Fig 3.

### Species-level discrimination by NA-SERS

Our final question of interest was the ability of the platform to discriminate among the 13 different species analyzed in this study. However, PLS-DA classification performance diminishes as the number of classes in question increases, due to the underlying algorithms applied by the modeling, and as such, attempts to classify all 13 species within a single PLS-DA model failed to yield statistically significant accuracy. In order to overcome this limitation and address the question in a clinically relevant classification system, individual pair-wise PLS-DA modeling was employed. For this analysis, we chose to focus on the nine other human commensal and pathogenic mycoplasma species most closely related phylogentically to *M*. *pneumoniae*. Individual PLS-DA models were built for each of the nine mycoplasma species to distinguish among three categories: the growth medium control (1), *M*. *pneumoniae* (2), or the mycoplasma species in question. Each model contained a total of n = 30 spectra, with 10 spectra representing each category. Furthermore, for each category eight of the 10 spectra were of known sample class, whereas two out of the 10 spectra were treated as unknowns and classified based on their resemblance to category 1, 2, or 3 spectra. Cross-validation of the prediction capability for each model was done using a Venetian blinds algorithm with five data splits. Models were designed this way to simulate the prediction of a potential unknown clinical sample as mycoplasma-negative, *M*. *pneumoniae*-positive, or positive for one of the other human commensal or pathogenic mycoplasma species. The cross-validated sensitivities and specificities for all nine models are given in Table 5. Overall, the cross-validated sensitivity and specificity was 90% or greater for all nine models, which is very promising for further application of NA-SERS for species-level discrimination and prediction.

**Table 5:** Cross-validated PLS-DA modeling statistics for the prediction performance for species discrimination between *M*. *pneumoniae* and nine other human commensal and pathogenic mycoplasma species individually.

Isolate	CV Sensitivity	CV Specificity	CV Class Error
1: GMC	1	1	0
2: M129	1	1	0
3: *M*. *amphoriforme*	1	0.95	0.025
1: GMC	1	1	0
2: M129	1	1	0
3: *M*. *fermentans*	1	1	0
1: GMC	1	1	0
2: M129	1	1	0
3: *M*. *genitalium*	1	1	0
1: GMC	0.9	1	0.05
2: M129	1	1	0
3: *M*. *hominis*	1	1	0
1: GMC	1	1	0
2: M129	1	1	0
3: *M*. *orale*	1	1	0
1: GMC	1	1	0
2: M129	1	0.95	0.025
3: *M*. *penetrans*	1	1	0
1: GMC	1	1	0
2: M129	1	1	0
3: *M*. *pirum*	1	1	0
1: GMC	1	1	0
2: M129	1	1	0
3: *M*. *salivarium*	1	0.9	0.05
1: GMC	1	1	0
2: M129	1	1	0
3: *M*. *spermatophilum*	1	0.95	0.025

For species discrimination, three categories were incorporated to cross-validate the model: either category 1: growth medium control (GMC); category 2: *M*. *pneumoniae* (M129); or category 3: other mycoplasma species in question. Eight spectra from each category were of known class and two spectra from each category were treated as unknowns to generate a cross-validated prediction model. Cross-validated sensitivity, specificity, and class error were based on the classification prediction score for each category.

## Conclusions

*M*. *pneumoniae* is a significant human respiratory tract pathogen in both incidence and public health impact, but diagnostic strategies are complicated by the atypical and complex presentation of disease, non-descript symptoms, the requirement for separate tests for detection and genotyping, and the challenges posed by direct culture. Serologic testing was historically the gold standard for diagnosis but suffers from severe limitations that make it both unreliable and impractical for rapid detection. Advances in qPCR technologies have overcome many issues with sensitivity and reliability, but the cost of reagents and requirement for technical expertise are still high, and independent tests must be done for detection and genotyping, limiting diagnosis by qPCR to hospital or advanced laboratory facilities and making it impractical for point-of-care use.

We previously established that this NA-SERS biosensing platform is capable of statistically significant detection of *M*. *pneumoniae* in true and simulated throat swabs and has a qualitative endpoint of detection for *M*. *pneumoniae* of < 1 cell/μl, a sensitivity exceeding that of qPCR [13, 31]. Here, NA-SERS showed statistically significant specificity for *M*. *pneumoniae* detection regardless of clinical isolate origin, year of isolation, macrolide susceptibility phenotype, or strain type, and was also able to distinguish all *M*. *pneumoniae* clinical isolates and control strains from 12 other human commensal and pathogenic *Mollicutes* species. Furthermore, NA-SERS discriminated between the two major strain types of *M*. *pneumoniae* with a high degree of statistically significant accuracy and correctly identified variant strains as different from the two major genotypes. Most importantly, NA-SERS was capable of detecting and strain-typing *M*. *pneumoniae* within a single test and thus has the potential to facilitate tracking epidemiological trends, such as type-switching and outbreak periodicity [12]. Studies with clinical samples are ongoing, and the effect of the presence of a clinically relevant sample background, for example from a patient’s throat swab, on the ability of this platform to identify strain types or distinguish *M*. *pneumoniae* from other human commensal and pathogenic *Mollicutes* species remains to be determined. Furthermore, assessment of alternative methods of chemometric analysis to account for the increased number of classes and modeling complexity for species-level discrimination by NA-SERS is necessary. In addition, future clinical application of this technology will require collection of a larger spectral library of isolates and background controls. However, from a point-of-care clinical standpoint, the ability to detect *M*. *pneumoniae* rapidly is critical to informing appropriate treatment regimens consistent with the responsible use of antimicrobials. This feature is underscored by the availability of handheld Raman instruments having the potential for point-of-care use [52–54]. In combination with the minimal sample preparation requirements and expedient detection, NA-SERS shows great promise as a platform for future application to point-of-care *M*. *pneumoniae* diagnostics.
